# Role of IKK/NF-κB Signaling in Extinction of Conditioned Place Aversion Memory in Rats

**DOI:** 10.1371/journal.pone.0039696

**Published:** 2012-06-26

**Authors:** Cheng-Hao Yang, Xiang-Ming Liu, Ji-Jian Si, Hai-Shui Shi, Yan-Xue Xue, Jian-Feng Liu, Yi-Xiao Luo, Chen Chen, Peng Li, Jian-Li Yang, Ping Wu, Lin Lu

**Affiliations:** 1 Tianjin Medical University, Tianjin, China; 2 Department of Thoracic Oncology, Tianjin Cancer Institute and Hospital, Tianjin Medical University, Tianjin, China; 3 National Institute on Drug Dependence, Peking University, Beijing, China; 4 Tianjin Institute of Mental Health, Tianjin Mental Health Center, Tianjin, China; Florida State University, United States of America

## Abstract

The inhibitor κB protein kinase/nuclear factor κB (IKK/NF-κB) signaling pathway is critical for synaptic plasticity. However, the role of IKK/NF-κB in drug withdrawal-associated conditioned place aversion (CPA) memory is unknown. Here, we showed that inhibition of IKK/NF-κB by sulphasalazine (SSZ; 10 mM, i.c.v.) selectively blocked the extinction but not acquisition or expression of morphine-induced CPA in rats. The blockade of CPA extinction induced by SSZ was abolished by sodium butyrate, an inhibitor of histone deacetylase. Thus, the IKK/NF-κB signaling pathway might play a critical role in the extinction of morphine-induced CPA in rats and might be a potential pharmacotherapy target for opiate addiction.

## Introduction

Opiate addiction is a chronic relapsing disorder characterized by compulsive drug seeking and taking motivated by the desire not only to experience the hedonic effects of the drug but also to avoid the aversive consequences of drug withdrawal [1], [2], [3]. Drug withdrawal-induced aversive memories have been proposed to play an important role in the compulsivity associated with drug seeking and taking [4], [5], [6]. Morphine withdrawal-induced conditioned place aversion (CPA) memory has been widely used in animals to investigate the negative consequences by drug withdrawal [7], [8]. Behavioral extinction training effectively abolished the expression of morphine-induced CPA [7]. Interestingly, recent evidence suggested that extinction training as a new memory did not erase but rather suppressed the conditioned response [9], [10]. The exploration of the underlying mechanism is important to prevent relapse induced by anhedonia and the aversive consequences of drug withdrawal.

Previous studies demonstrated the role of the transcription factor nuclear factor κB (NF-κB) family, which consists of five members [11], [12], in synaptic plasticity and long-term memory [12], [13], [14], [15], [16], [17], [18]. Specifically, c-Rel, an NF-κB family transcription factor, is required for hippocampal long-term synaptic plasticity and contextual fear memory formation [12]. Activation of the transcription factor NF-κB by retrieval is required for long-term memory reconsolidation [19], [20]. NF-κB signaling played an important role in the extinction of long-term memory in a context-signal memory model in the crab *Chasmagnathus* [21]. Inhibition of NF-κB prior to the extinction session prevented spontaneous recovery. Our recent study indicated that NF-κB inhibition impaired the reconsolidation of morphine-associated reward memory in rats [22]. To produce its function, NF-κB is translocated into the nucleus and then binds to the promoter region of target genes [23]. NF-κB was inactivated when combined with inhibitor κB (IκB) proteins, which could be phosphorylated by the IκB kinase (IKK) complex [24], [25] and then undergo proteolytic degradation. The IKK complex was recently shown to regulate chromatin structure. The histone deacetylase (HDAC) inhibitor sodium butyrate (NaB) blocked the effect of IKK/NF-κB inhibition on memory reconsolidation [20]. However, the role of IKK/NF-κB signaling in the extinction of drug withdrawal-associated aversive memories is still unknown.

## Results

### Inhibition of IKK/NF-κB Pathway by SSZ Suppressed the Extinction of Morphine Withdrawal-associated Aversive Memory

**Figure 1:**
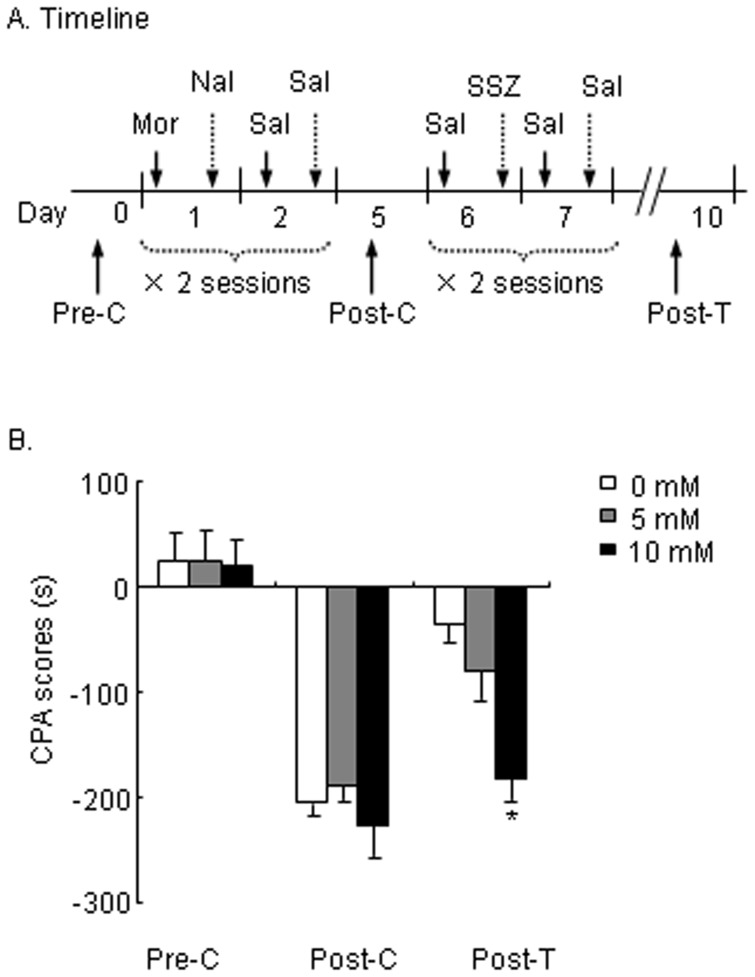
Sulphasalazine (SSZ) suppressed the extinction of morphine withdrawal-associated aversive memory. (A) Timeline of experimental procedure. (B) Infusion of the IKK/NF-κB pathway blocker SSZ (0, 5, and 10 mM) into the cerebral ventricles 20 min before extinction training suppressed the extinction of morphine withdrawal-associated aversion memory. The data are expressed as the mean ± SEM CPA score in seconds (time spent in the drug-paired chamber minus time spent in the saline-paired chamber) during the CPA tests. *p<0.01, different from control group (0 mM SSZ treatment group). Mor, morphine; Nal, naloxone; Sal, saline; Pre-C, pre-conditioning; Post-C, post-conditioning; Post-T, post-treatment.

To investigate the effects of IKK/NF-κB signaling inhibition on the extinction of morphine-associated aversive memory (Fig. 1A), four groups of rats (*n* = 8−13 per group) which acquired CPA were infused with sulphasalazine (SSZ; 0, 5, and 10 mM, i.c.v.) 20 min before extinction training [19], [21] on day 6 and day 8. The rats were tested for the expression of CPA (Post-T) 24 h after the second SSZ infusion. A repeated-measures analysis of variance (ANOVA) was conducted on CPA scores, with SSZ dose (0, 5, and 10) as the between-subjects factor and test condition (Pre-C, Post-C, and Post-T) as the within-subjects factor. The results revealed significant effects of SSZ dose (*F*_2,29_ *= *4.090, *p*<0.05) and test condition (*F*_2,58_ = 91.97, *p*<0.001) and a treatment × test condition interaction (*F*_4,58_ = 4.399, *p*<0.01; Fig. 1B). These results indicate that SSZ-induced (10 mM, i.c.v.) inhibition of IKK/NF-κB suppressed the extinction of morphine withdrawal-associated aversive memory.

### Sulfasalazine had no Effect on the Acquisition or Expression of Morphine Withdrawal-associated Aversive Memory

**Figure 2:**
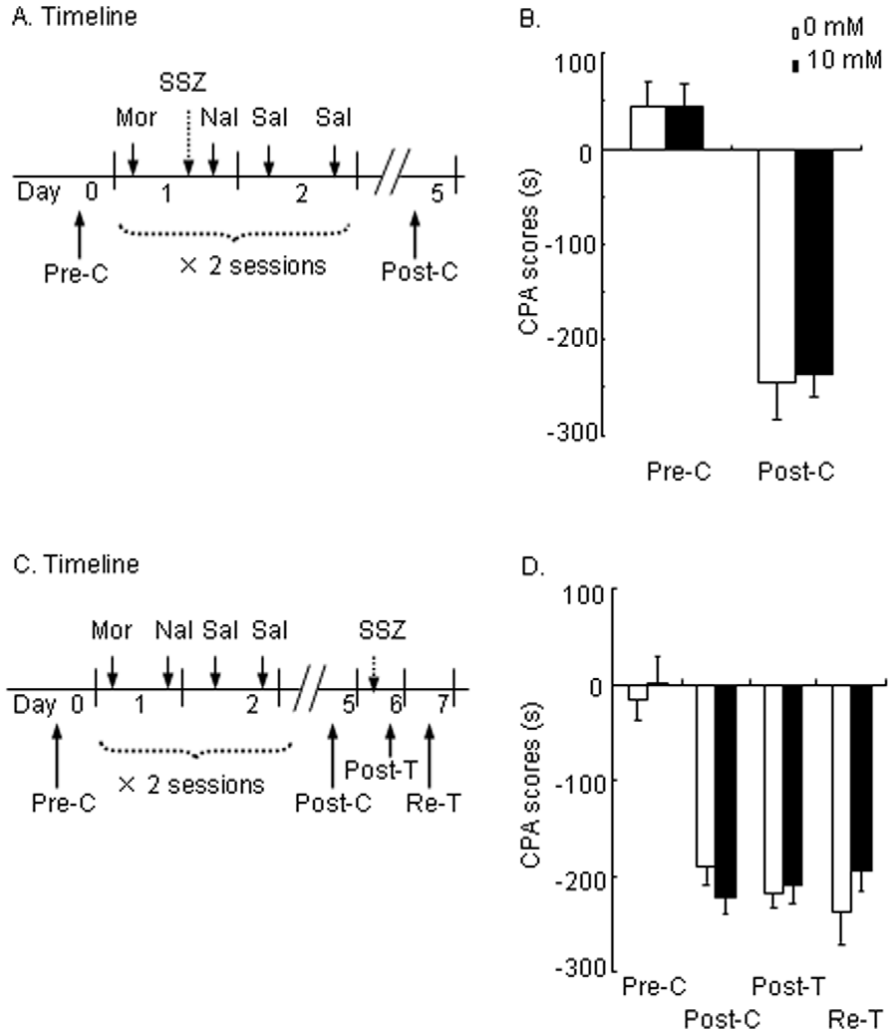
Sulphasalazine (SSZ) had no effect on the acquisition or expression of morphine withdrawal-associated aversive memory. (A) Timeline of experimental procedure. (B) Infusion of SSZ (0 and 10 mM) into the cerebral ventricles 20 min before CPA training had no effect on the acquisition of morphine withdrawal-associated aversive memory (*p*>0.05). (C) Timeline of experiment procedure. (D) Infusion of SSZ (0 and 10 mM) into the cerebral ventricles 20 min before CPA training had no effects on the expression of morphine withdrawal-associated aversive memory (*p*>0.05). The data are expressed as the mean ± SEM CPA score in seconds (time spent in the drug-paired chamber minus time spent in the saline-paired chamber) during the CPA tests. Mor, morphine; Nal, naloxone; Sal, saline; Pre-C, pre-conditioning; Post-C, post-conditioning; Post-T, post-treatment; Re-T, retest.

As extinction has been suggested to be a new memory, we next tested whether SSZ affects the acquisition of morphine withdrawal-associated CPA. After recovery from surgery, the rats were randomly divided into two groups (*n* = 9 per group) according to the Pre-C test. The rats were then trained for morphine-induced CPA for 4 days, during which SSZ (10 mM, i.c.v.) was infused 20 min before each naloxone injection (Fig. 2A). The expression of CPA was tested on day 5 (Post-C). A repeated-measures ANOVA conducted on CPA scores, with SSZ dose (0 and 10 mM) as the between-subjects factor and test condition (Pre-C and Post-C) as the within-subjects factor. The results revealed a significant effect of test condition (*F*_1,16_ = 166.922, *p*<0.001) but no effect of treatment (*F*_1,16_ = 0.024, *p*>0.05) and no treatment × test condition interaction (*F*_1,16_ = 0.039, *p*>0.05; Fig. 2B), indicating that IKK/NF-κB activity was not involved in the acquisition of morphine withdrawal-associated CPA.

The extinction of morphine-induced CPA was blocked by SSZ infused 20 min before the training session, which might be attributable to the enhanced expression of CPA but not blockade of extinction. To exclude this possibility, we tested whether SSZ affects the expression of CPA. After recovery from surgery, two groups of rats (*n* = 8 per group) were trained for morphine withdrawal-associated aversive memory (Fig. 2C). The rats were tested (Post-T) for the expression of CPA 20 min after SSZ infusion (0 and 10 mM, i.c.v.). Twenty-four hours later, the rats were tested again (Re-Test) to exclude the possible delayed effect of SSZ. A repeated-measures ANOVA was conducted on CPA scores, with SSZ dose (0 and 10 mM) as the between-subjects factor and test condition (Pre-C, Post-C, Post-T, and Re-Test) as the within-subjects factor. The results revealed no significant effect of treatment (*F*_1,14_ = 0.284, *p*>0.05) and no treatment × test condition interaction (*F*_3,42_ = 1.148, *p*>0.05; Fig. 2D), suggesting that IKK/NF-κB activity was not involved in the expression of morphine withdrawal-associated aversive memory.

### NaB Abolished the Effect of SSZ on Extinction of Morphine Withdrawal-associated Aversive Memory

**Figure 3:**
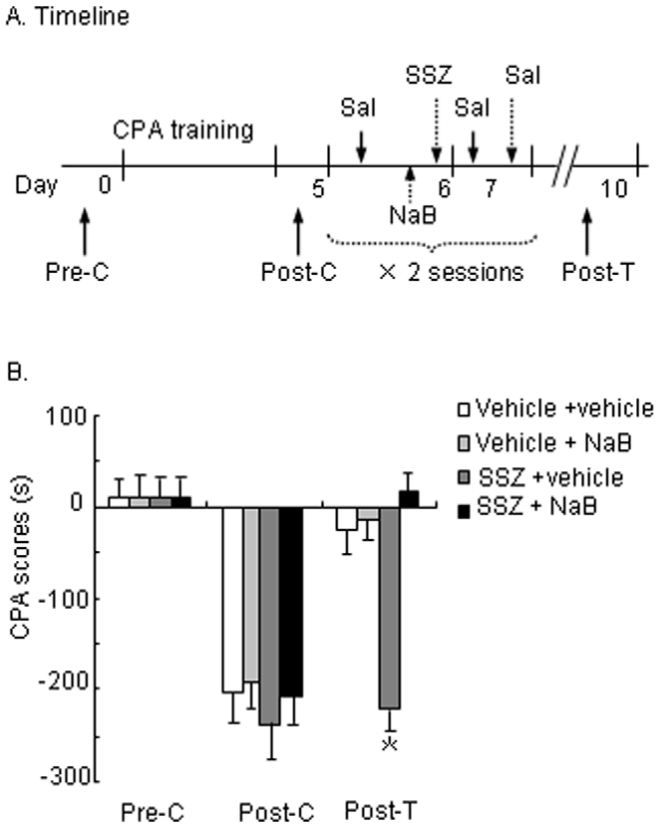
Sodium butyrate (NaB) abolished the SSZ-induced inhibition of extinction of morphine withdrawal-associated aversive memory. (A) Timeline of experiment procedure. (B) Systemic pretreatment with NaB abolished the SSZ-induced inhibition of extinction of morphine withdrawal-associated aversive memory. The data are expressed as the mean ± SEM CPA score in seconds (time spent in the drug-paired chamber minus time spent in the saline-paired chamber) during the CPA tests. *p<0.01, different from control group (0 mM SSZ treatment group). Sal, saline; SSZ, sulfasalazine; Pre-C, pre-conditioning; Post-C, post-conditioning; Post-T, post-treatment.

Activated IKK/NF-κB signaling affects target gene expression regulated by HDAC activity. We determined whether NaB, an HDAC inhibitor, abolishes the effect of SSZ on the extinction of CPA. After recovery from surgery, the rats were randomly divided into four groups (*n* = 9−10 per group) after the establishment of CPA (Post-C; Fig. 3A). NaB (0 and 1.2 g/kg, i.p.) [20], [22] was administered 40 min before SSZ infusion (0 and 10 mM, i.c.v.). Twenty minutes after SSZ infusion, extinction training was conducted. A repeated-measures ANOVA was conducted on CPA scores, with SSZ dose (0 and 10 mM) and NaB dose (0 and 1.2 g/kg) as the between-subjects factors and test condition (Pre-C, Post-C, and Post-T) as the within-subjects factor. The results revealed significant effects of test condition (*F*_2,72_ = 90.490, *p*<0.001) and a NaB dose × SSZ dose × test condition interaction (*F*_2.72_ = 7.175, *p*<0.01; Fig. 3B). Conditioned place aversion scores in the rats that received both SSZ and NaB significantly decreased 24 h after extinction, indicating that NaB abolished the effect of SSZ on the extinction of CPA.

### Sulfasalazine had no Effects on Locomotor Activity or Reward Per Se

**Figure 4:**
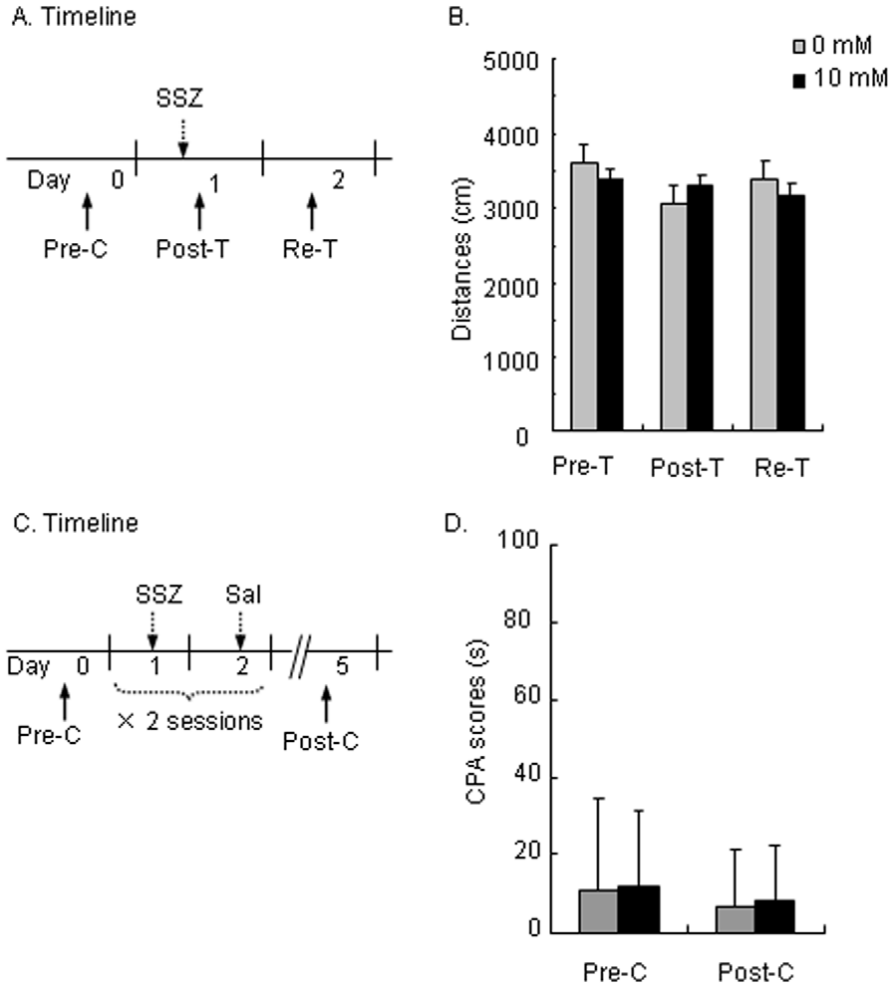
Sulfasalazine had no effect on locomotor activity and no aversive or rewarding effects. (A) Timeline of effects of SSZ on locomotor activity. Locomotor activity was tested 20 min (Post-T) and 24 h (Re-T) after SSZ administration (10 mM) for 5 min. (B) Sulfasalazine (10 mM) did not affect locomotor activity 20 min or 24 h after infusion. (C) Timeline of SSZ-induced conditioned place preference/aversive procedure. A double 2-day-session training procedure was used to test the aversive or rewarding effects of SSZ. (D) Sulfasalazine did not induce any aversion to or preference for the drug-paired chamber (CPA score), indicating that SSZ had no aversive or rewarding effects. The data are expressed as mean ± SEM. Pre-T, pre-treatment; Post-T, post-treatment; Re-T, re-test.

To determine that the effect of SSZ on the extinction of morphine-induced CPA was not attributable to the possible side effects of SSZ (e.g., psychoactive actions), we investigated the effect of SSZ on locomotor activity and place preference in rats. Two groups of rats (*n* = 7−8 per group) were tested for changes in locomotor activity 20 min and 24 h after intracranial administration of SSZ (Fig. 4A). A repeated-measures ANOVA was used to analyze locomotor activity, with SSZ dose (0 and 10 mM) as the between-subjects factor and Test Condition (pretreatment, post-treatment, and re-test) as the within-subjects factor. The analysis revealed no significant effects of SSZ (*F*_1,13_ = 0.066, *p*>0.1) and no SSZ × Test Condition interaction (*F*_2,26_ = 1.383, *p*>0.1). These results suggest that the inhibition of IKK/NF-κB activity did not affect locomotor activity in rats (Fig. 4B).

To exclude the possibility that SSZ inhibited the extinction of CPA through a possible aversive effect, two groups of rats were used to determine whether SSZ has any aversive or rewarding effects *per se* in a conditioned place preference test (Fig. 4C). In this experiment, the rats underwent SSZ or vehicle conditioning for two sessions and were tested for place preference on Day 5. A repeated-measures ANOVA was conducted on CPA scores, with SSZ dose (0 and 10 mM) as the between-subjects factor and Test Condition (preconditioning and post-conditioning) as the within-subjects factor. The analysis revealed no significant effect of SSZ (*F*_1,13_ = 0.001, *p*>0.5) and no SSZ × Test Condition interaction (*F*_1,13_<0.01, *p*>0.5). These results suggest that the rats showed no preference for the SSZ-paired chambers in the post-conditioning test on Day 5, indicating that SSZ administered intracerebroventricularly had no aversive or rewarding effects (Fig. 4D).

## Discussion

Collectively, the present study determined the role of the IKK/NF-κB signaling pathway in morphine withdrawal-associated CPA. We found that blockade of the IKK/NF-κB signaling pathway by intracerebroventricular SSZ infusion selectively suppressed the extinction but not acquisition or expression of morphine withdrawal-associated CPA, which was blocked by pretreatment with an HDAC inhibitor, sodium butyrate.

Extinction training has been suggested to form a new memory, resulting in the loss of the response to cues associated with emotional stimuli [26]. Extensive research has focused on discovering the mechanism of extinction, and has provided evidence of the involvement of the *N*-methyl-D-aspartate receptor (NMDAR) [27], [28], [29]. Activation of NMDARs by glutamate or acute stress has been shown to activate NF-κB in several brain structures [30], [31], [32], [33]. The signaling pathway that underlies the participation of NF-κB in memory formation, including protein kinase A and its substrate cyclic adenosine monophosphate response element binding protein, may play a critical role in the extinction of morphine-induced CPA [34]. Moreover, NF-κB can affect the growth and morphology of axons and dendrites to affect the extinction of CPA [35]. Conditioned place aversion is a hippocampus-dependent memory, and NF-κB may also affect hippocampal neurogenesis to participate in the morphine-induced CPA extinction process [36]. A context-signal memory model in the crab *Chasmagnathus* showed that NF-κB inhibition facilitated extinction. The activation or inhibition of NF-κB was dependent on the length of exposure to the training context, indicating that NF-κB was involved in reconsolidation, and NF-κB inhibition was involved in extinction [21]. de la Fuente et al. found that NF-κB was required for fear memory reconsolidation, whereas NF-κB inhibition in the mouse hippocampus enhanced memory extinction [37]. Interestingly, the results of our experiments showed that SSZ, an inhibitor of the IKK/NF-κB signaling pathway, suppressed the extinction of morphine-induced CPA. However, as the limitation of intracerebroventricular infusion of SSZ used in the present experiment to inhibit NF-κB, we could not identify the specific brain subregion where NF-κB played the important role in the extinction of morphine CPA.

These contradictory results might indicate that the role of NF-κB in extinction might be memory type-dependent. We propose the following explanations. First, opioid receptors are involved in morphine withdrawal-associated aversive memory, so the primarily activated downstream signaling pathways might be different with the other types of aversive memory models (e.g., fear memory). Second, evidence has shown that the facilitatory effect of NF-κB is necessary in memory consolidation and reconsolidation in crabs and mice [19], [20], [38], [39]. As extinction has been suggested to be a new type of memory, NF-κB might facilitate the consolidation of CPA extinction.

Many studies have shown that IKKα could be translocated into the nucleus to mediate NF-κB-dependent and -independent gene expression [40], [41], [42]. SSZ is a well-established antiinflammatory drug that has been shown to specifically and potently inhibit the activation of NF-κB [43]. The suppression of NF-κB activity by SSZ is mediated by direct inhibition of IκB kinases α and β. It prevents the phosphorylation and subsequent degradation of the inhibitory NF-κB IκB β subunit [44], blocks NF-κB translocation into the nucleus, and suppresses the regulation of chromatin structure by IKKα [20].

Sodium butyrate is a noncompetitive inhibitor of HDAC activity [45]. It can halt DNA synthesis, arrest cell proliferation, alter cell morphology, and regulate gene expression in cultured cells [46] by increasing histone acetylation [47], [48]. It has also been implicated in depression, anxiety [49], and long-term memory formation [50] through epigenetic mechanisms. A recent study reported that intrahippocampal and intra-medial prefrontal cortex administration of NaB following weak extinction induced behavioral extinction, indicating that increasing histone acetylation in the hippocampus-infralimbic network enhances fear extinction [51]. In the present study, systemic administration of NaB blocked the SSZ-induced inhibition of the IKK/NF-κB signaling pathway, which might involve two possible mechanisms: (*i*) directly regulating chromatin remodeling by enhancing histone acetylation and (*ii*) indirectly regulating gene expression by enhancing NF-κB acetylation and NF-κB DNA binding activity. Additionally, NaB enhanced memory retention even after weak training [52]. Interestingly, in the present study, NaB did not enhance the extinction of morphine-induced CPA but abolished the SSZ-induced inhibition of CPA extinction. We suggest that the lack of NaB-induced enhancement of CPA extinction might be attributable to a ceiling effect because CPA scores in the control group after extinction decreased to baseline levels. Moreover, our results suggest that NF-κB requires the activity of HDAC to exert its function, specifically to induce the target gene expression engaged in the extinction of morphine-induced CPA.

The present study has several limitations, the most significant of which is that we did not present molecular endpoints to substantiate the theorized molecular mechanisms of the effects on extinction. Future work that investigates the downstream target gene expression induced by NF-κB would be helpful to elucidate the mechanism of extinction of morphine withdrawal-associated CPA memory.

In summary, the present study demonstrated that the IKK/NF-κB signaling pathway is involved in the extinction of morphine withdrawal-associated aversive memory. Inhibition of the IKK/NF-κB signaling cascade abolished the extinction of morphine withdrawal-associated CPA, which was reversed by systemic pretreatment with NaB, an HDAC inhibitor. IKK/NF-κB activity was shown to be involved in the extinction but not acquisition or expression of morphine withdrawal-associated CPA, suggesting that the extinction of morphine withdrawal-associated CPA depends on IKK/NF-κB activity. However, further studies are needed to clearly delineate the mechanisms that underlie the role of the IKK/NF-κB signaling cascade in the extinction of morphine-associated aversive memory. Nevertheless, these results suggest that IKK/NF-κB may be a potential target for opioid relapse prevention.

## Materials and Methods

### Animals

Sprague-Dawley male rats, weighing 220–240 g, were obtained from the Laboratory Animal Center, Peking University Health Science Center. The rats were housed in groups of five in a temperature (23±2°C) and humidity (50±5%) controlled animal facility with *ad libitum* access to food and water. The rats were kept on a reverse 12 hours light/dark cycle. All experimental procedures were performed in accordance with the National Institutes of Health Guide for the Care and Use of Laboratory Animals and were approved by Biomedical Ethics Committee of Peking University of animal use and protection.

### Surgery

During surgery, each rat was implanted with a 23-gauge single guide cannula (Plastics One, Roanoke, VA, USA) from which the injector extended 1 mm for placement in the left or right lateral ventricle after anesthetizing with sodium pentobarbital (50 mg/kg, i.p.). The stereotaxic coordinates were the following, measured from the tip of the cannula guide: 1.0 mm from bregma, ±1.2 mm lateral from the midline, and 3.5 mm from dura [20]. Animals were habituated to dummy cannula removal and given 7 days of recovery and handling before the start of behavioral training.

### Drugs

Sodium butyrate and SSZ were purchased from Sigma (St. Louis, MO, USA). Sulfasalazine was freshly dissolved in saline solution with 10 mM HEPES (pH 7.6) plus 20% dimethyl sulfoxide (final pH 7.6) [20]. Sodium butyrate was dissolved in saline solution. For the experiment that investigated the effects of NaB on CPA memory extinction, the rats were injected with 1.2 g/kg NaB 1 h prior to extinction training [20], [22]. Morphine hydrochloride was obtained from Qinghai Pharmaceuticals (Qinghai, China). Naloxone hydrochloride was purchased from Tocris Bioscience (Bristol, UK). All of the drugs were freshly prepared before the experiments. Naloxone and morphine were dissolved in saline (Sal, 0.9% NaCl), and both injections were administered subcutaneously in a 1 ml/kg injection volume that was selected based on our previous studies [7].

### Intracranial Injections

Injections of SSZ (0, 5, and 10 mM) into the lateral ventricle were performed according to our previous study [22] using Hamilton syringes connected to 30-gauge injectors (Plastics One). Sulfasalazine was administered at rate of 1 µl/min for 1 min. After the injection, the needle was kept in place for another 1 min to allow for complete diffusion [23].

### Naloxone-induced Conditioned Place Aversion

The CPA apparatus used in this study consisted of five identical three-chamber polyvinylchloride (PVC) boxes [53], [54]. Two large side chambers (27.9 cm length ×21.0 cm width ×20.9 cm height) were separated by a smaller chamber (12.1 cm length ×21.0 cm width ×20.9 cm height with a smooth PVC floor). The two larger chambers differed in their floor texture (i.e., bar or grid, respectively) and provided distinct contexts that were paired with morphine/naloxone or saline injections. Three distinct chambers were separated by manual guillotine doors that were removed during the test [55], [56].

The CPA training procedure was an unbiased, counterbalanced protocol that was similar to the one used in previous experiments [57], [58]. The protocol consisted of three phases: preconditioning, conditioning, and test. In the preconditioning phase (Day 0), the rats were placed in the middle chamber and allowed to shuttle between the three chambers in the apparatus for 15 min. The time spent in each chamber was recorded (Pre-C), and the rats that showed a strong unconditioned aversion (>180 s) for either chamber were eliminated from the study [57]. Twenty-four hours later, a two-session CPA conditioning session was conducted. Each session included 2 days. On the first day (Day 1 and Day 3), the rats were injected with morphine (5 mg/kg, s.c.). Four hours later, they were injected with naloxone (0.3 mg/kg, s.c.) and confined to the designated chamber (i.e., drug-paired chamber) for 30 min. On the second day (Day 2 and Day 4), the rats were injected with saline (1 ml/kg, s.c.). Four hours later, they were given saline (1 ml/kg, s.c.) and then confined to the chamber (i.e., saline-paired chamber) opposite to the chamber on the first day for 30 min. On Day 5, the expression of naloxone-precipitated CPA was tested in a drug-free state. The testing procedure was the same as the preconditioning test. The CPA score was defined as the time (in seconds) spent in the drug-paired chamber minus the time spent in the saline-paired chamber during the test phase [7], [57].

In the experiment that explored the effects of IKK/NF-κB signaling inhibition on the acquisition of CPA, two groups of rats (*n* = 9 per group) were administered SSZ (0 or 10 mM in 5 µl of vehicle, i.c.v.) 20 min before naloxone injection during every CPA training session (Fig. 2A). In the experiment that examined the effects of IKK/NF-κB signaling inhibition on the expression of CPA, two groups of rats (*n*  = 8 per group) that showed morphine withdrawal-associated aversive memory on Day 5 were administered SSZ (0 or 10 mM in 5 µl of vehicle, i.c.v.) 20 min before the CPA expression test (Post-T) on Day 6 (Fig. 2C). The rats were tested again on Day 7 (Re-test) to exclude possible delay effects.

### Conditioned Place Aversion Extinction Training

The CPA extinction was performed for 4 consecutive days (Day 6–9) under the conditions that was identical to CPA training (restrictive extinction), with the exception that naloxone and morphine were replaced by saline. The CPA test (Day 10) was performed 24 hours after the second extinction training session.

In the experiment that investigated the effects of IKK/NF-κB signaling inhibition on the extinction of CPA (Fig. 1A), three groups of rats (*n* = 8−13 per group) that had acquired CPA were given a SSZ microinjection (0, 5, or 10 mM in 5 µl of vehicle, i.c.v.) 20 min before each extinction training session (Day 6 and Day 8) [19], [21]. Twenty-four hours after extinction training, the rats were tested for the expression of CPA (Day 10).

In the experiment that determined the possible role of HDAC activity in the NF-κB-dependent extinction of CPA (Fig. 3A), four groups of CPA-conditioned rats were administered NaB (0 or 1.2 g/kg, i.p.) and SSZ (0 or 10 mM in 5 µl of vehicle, i.c.v.) 1 h and 20 min before the naloxone injection during every extinction training, respectively. The rats were also tested for the expression of CPA 24 h later on Day 10.

### Sulfasalazine-induced Conditioned Place Preference/Aversion

To exclude the possibility that SSZ has aversive or rewarding effects *per se*, two groups of rats were tested for SSZ-induced conditioned place preference (Fig. 4C). The conditioned place preference/aversion procedure included preconditioning (Pre-C), conditioning, and post-conditioning (Post-C) phases. On the first day (Day 0), the rats were tested for baseline preference/aversion for 15 min. In the following phase (Day 1–4), the rats received alternate injections of SSZ (10 mM in 0.5 µl, i.c.v.) and vehicle (0.5 µl, i.c.v.) 20 min before being confined to the corresponding chamber for 30 min per day for 4 days. On Day 5, the rats were tested for SSZ-induced conditioned place preference/aversion under conditions identical to the Pre-C test.

### Effect of SSZ on Locomotor Activity

We used two groups of rats (*n* = 7−8 per group) to determine whether SSZ affects locomotor activity in rats (Fig. 4A). The rats were placed in the testing chamber for 5 min to habituate them to the procedures used in the test on Day 0. Twenty-four hours later (Day 1), the rats were tested for 5 min in the testing chamber 20 min (Post-T) after SSZ treatment (0 or 10 mM in 5 µl of vehicle, i.c.v.). The rats were tested again on Day 2 to exclude possible delay effects.

An automated video tracking system (DigBehv-LM4, Shanghai Jiliang Software Technology Co. Ltd, Shanghai, China), including eight identical clear Plexiglas chambers (40×40×65 cm) equipped with a monochrome video camera on top, was used to measure locomotor activity. A computer was connected to the chambers to record the video files. DigBev Analysis Software was used to analyze the video files. Locomotor activity is expressed as the total distance traveled during the 5 min test.

### Statistical Analysis

All of the data are expressed as mean ± SEM. The statistical analysis was performed using ANOVA with appropriate between- and within-subjects factors for the different experiments (see Results). *Post hoc* analyses of significant effects in the ANOVA were performed using the Tukey test. Values of *p*<0.05 were considered statistically significant.
